# WJNM with WARMTH: A Driving Force in Promoting Global Nuclear Medicine

**DOI:** 10.1055/s-0046-1820111

**Published:** 2026-04-09

**Authors:** Kalevi Kairemo, Akram Al-Ibraheem

**Affiliations:** 1 Department of Radiotheranostics, Docrates Cancer Center, Helsinki, Finland; 2 Department of Nuclear Medicine, King Hussein Cancer Center (KHCC), School of Medicine, University of Jordan, Amman, Jordan

*World Journal of Nuclear Medicine* (WJNM) is the official journal of the World Association of Radiopharmaceutical and Molecular Therapy (WARMTH).

WJNM is experiencing a phase of continuity and strategic progression, ensuring sustained publication standards while advancing the journal's future direction. This issue reflects a coordinated effort to maintain editorial consistency and timely dissemination of scientific content. The scientific periodical WJNM is characterized by a large number of case reports and is now actively transitioning toward a stronger emphasis on high-quality original research and authoritative review articles. The current issue represents a step toward a more balanced composition of article types, although this balance is not yet fully achieved, as many included manuscripts were submitted before this editorial direction was introduced. The WJNM remains committed as a truly global platform, with continued emphasis on geographical diversity and inclusivity in scientific contributions.

WARMTH, as a voluntary nonprofit organization dedicated to advancing science and education in radiopharmaceutical therapy, continues to advance science and education of radiopharmaceutical therapies for the benefit of public health and humanity and is willing to work toward worldwide access to these theragnostic procedures. 1

Radiotheranostics is undergoing a paradigm shift, evolving from a predominantly investigational discipline into a central pillar of precision oncology. This transformation is driven by advances in molecular imaging, targeted radionuclide therapy, and patient-specific dosimetry, enabling more personalized and effective treatment strategies. 2 3 4 5 In this context, WJNM aims not only to disseminate scientific knowledge but also to facilitate clinical translation, standardization of practice, and global accessibility of theragnostic applications.

During the year 2025, many collaborative larger meetings were held. The main congress of WARMTH, the 20th International Conference on Radionuclide Therapy held from November 6 to 9, 2025 in Cyprus, was arranged in collaboration with the Cyprus Society of Nuclear Medicine. 6 An International Symposium of Radiopharmaceutical Therapy (ISRT) was held from April 10 to 12, 2025 in Baku, Azerbaijan, together with the Azerbaijan Society of Nuclear Medicine. 1 Additionally, WARMTH actively participated in the ALASBIMN (Latin American Societies) meeting in March 2025 in Quintana Roo, Mexico, and WARMTH signed a collaboration agreement with ALASBIMN. At the Society of Nuclear Medicine and Molecular Imaging (SNMMI) meeting in June in New Orleans, WARMTH arranged its own session in collaboration with SNMMI. In the European Association of Nuclear Medicine (EANM) meeting in October 2025 in Barcelona, Spain, WARMTH was also present with its own booth.

This WJNM journal (ISSN 1450–1147) has an impact factor of 0.9, with clear strategic efforts underway to enhance its scientific impact and visibility. Recent initiatives, including thematic special issues and increased emphasis on original research, are expected to contribute to progressive improvement in journal metrics and indexing performance. At the end of November 2025, WARMTH elected new officials with ongoing efforts to further strengthen the editorial leadership structure of WJNM. The very first World Theragnostics Day (WTD) was celebrated on March 31, 2019 (the proclamation was published in the WJNM as a part of the Helsinki ISRT Abstracts 7 ). This year, WTD will be called Saul Hertz World Theragnostics Day and an online event will be held. For WTD 2026, WARMTH has organized a free online 1-hour interactive educational webinar—Expanding Horizons in PRRT—with two eminent speakers, addressing theragnostics of neuroblastoma and neuroendocrine tumors.

WARMTH's deepened collaboration with international societies such as IAEA, WFNMB, ICPO, SNMMI, EANM, AOFNMB, AANM, ARSNM, ALASBIMN, etc. 8 9 will continue. New proposals for local ISRTs from regional societies are always welcome, and at least abstracts of these events will be considered for publication in WJNM. In parallel, dedicated special issues on focused thematic areas are being developed to further enhance the journal's scientific impact. Collectively, these efforts aim to harmonize clinical practice, advance education, and support capacity building across diverse health care systems.
